# A novel DNA methylation signature to improve survival prediction of progression-free survival for testicular germ cell tumors

**DOI:** 10.1038/s41598-023-30957-6

**Published:** 2023-03-07

**Authors:** Feng Gao, Qiaoping Xu, Yingjun Jiang, Bingjun Lu

**Affiliations:** 1 Department of Urology, Hangzhou Hospital of Traditional Chinese Medicine, No. 453, Stadium Road, Xihu District, Hangzhou, 310000 China; 2 Department of Clinical Pharmacology, Key Laboratory of Clinical Cancer Pharmacology and Toxicology Research of Zhejiang Province, Affiliated Hangzhou First People’s Hospital, Cancer Center, Zhejiang University School of Medicine, Hangzhou, 310006 China

**Keywords:** Cancer, Computational biology and bioinformatics, Biomarkers, Risk factors

## Abstract

This study aimed to develop a nomogram for predicting the progression-free survival (PFS) of testicular germ cell tumors (TGCT) patients based on DNA methylation signature and clinicopathological characteristics. The DNA methylation profiles, transcriptome data, and clinical information of TGCT patients were obtained from the Cancer Genome Atlas (TCGA) database. Univariate Cox, lasso Cox, and stepwise multivariate Cox regression were applied to identify a prognostic CpG sites-derived risk signature. Differential expression analysis, functional enrichment analysis, immunoinfiltration analysis, chemotherapy sensitivity analysis, and clinical feature correlation analysis were performed to elucidate the differences among risk groups. A prognostic nomogram integrating CpG sites-derived risk signature and clinicopathological features was further established and evaluated likewise. A risk score model based on 7 CpG sites was developed and found to exhibit significant differences among different survival, staging, radiotherapy, and chemotherapy subgroups. There were 1452 differentially expressed genes between the high- and low-risk groups, with 666 being higher expressed and 786 being lower expressed. Genes highly expressed were significantly enriched in immune-related biological processes and related to T-cell differentiation pathways; meanwhile, down-regulated genes were significantly enriched in extracellular matrix tissue organization-related biological processes and involved in multiple signaling pathways such as PI3K-AKT. As compared with the low-risk group, patients in the high-risk group had decreased lymphocyte infiltration (including T-cell and B-cell) and increased macrophage infiltration (M2 macrophages). They also showed decreased sensitivity to etoposide and bleomycin chemotherapy. Three clusters were obtained by consensus clustering analysis based on the 7 CpG sites and showed distinct prognostic features, and the risk scores in each cluster were significantly different. Multivariate Cox regression analysis found that the risk scores, age, chemotherapy, and staging were independent prognostic factors of PFS of TGCT, and the results were used to formulate a nomogram model that was validated to have a C-index of 0.812. Decision curve analysis showed that the nomogram model was superior to other strategies in the prediction of PFS of TGCT. In this study, we successfully established CpG sites-derived risk signature, which might serve as a useful tool in the prediction of PFS, immunoinfiltration, and chemotherapy sensitivity for TGCT patients.

## Introduction

Testicular cancer is the most frequent type of malignancy in young men aged 15–34 years old, while testicular germ cell tumors (TGCT) account for 90–95% of all testicular cancers^1^. TCGT was histologically divided into seminoma and non-seminoma germ cell tumors^2^, and non-seminomas consist of either undifferentiated or differentiated histologic subtypes^3^. TGCT presents high sensitivity to first-line platinum-based chemotherapy and radiotherapy, and the majority of patients could achieve high cure rates^4^. However, approximately 15% of patients don't respond to the first-line treatment. This is particularly true for non-seminomas, which cannot be cured using the first-line approach and require salvage therapy^5^. Currently, serum biomarkers, such as alpha-fetoprotein (AFP), human chorionic gonadotropin (HCG), and lactate dehydrogenase (LDH), and the Tumor Node Metastasis (TNM) classification were used to assist to make treatment decisions for TGCT patients^6^. However, the defects of these markers include poor specificity for the follow-up and monitoring of TGCT, and cannot accurately reflect the progression of the disease^7^. Therefore, the development of reliable genetic prognostic biomarkers for TGCT, especially in high-risk group, is urgently needed.

DNA methylation is a typical epigenetic modification modulating gene transcription, and aberrant DNA methylation was reported to be closely associated with tumor progression^8^. Growing evidence demonstrated that DNA methylation is implicated in the initiation, development, and progression of human cancers and may serve as potential prognostic biomarker. For instance, in TGCT, a previous study suggested that DNA methylation profiling could serve as a tool for testicular germ cell tumor subtyping^9^. MGMT and CALCA promoter methylation predicted the worse prognosis of TGCT patients and could be used as new molecular markers of prognosis in TGCT^10^. However, these studies focused on a few specific genes were limited by small sample sizes and generally generated unstable predictive robustness. Recently, DNA methylation signatures were identified to predict recurrence risk based on the whole-genome methylation profiles from the TCGA database for a variety of cancers, including lung cancer^11^, thyroid papillary carcinoma^12^, and gastric cancer^13^.

In the current study, we aimed to identify the prognostic DNA methylation sites for TGCT patients by analyzing the whole-genome DNA methylation profiles retrieved from a public database, and established a risk model for progression-free survival (PFS) prediction by combining the prognostic DNA methylation signature and clinicopathological parameters of TGCT patients.

## Material and methods

### Data resource

The DNA methylation data and corresponding clinical data of TGCT patients were obtained from the Cancer Genome Atlas (TCGA, https://cancergenome.nih.gov/) database by using the R TCGAbiolinks package^14^. All DNA methylation data were generated from the Illumina Infinium Human Methylation 450 platform and the levels of DNA methylation were expressed as β values, and calculated as M/(M + U + 100). M and U represent the signal from methylated beads and unmethylated beads at the target CpG sites, respectively. The methylomic data from patients with complete clinicopathological information were selected. The most recent clinicopathological and follow-up information was obtained from the TCGA database on 6 January 2023, clinical information and methylation data of a total of 128 TGCT samples were downloaded and analyzed in this study, and the samples were randomly classified into training cohort (89 samples) and validation cohort (39 samples) at a ratio of 7:3. Prognostic DNA methylation signature was identified based on the training cohort data, and the evaluation of the predictive ability was performed on the basis of the validation cohort data. Progression-free survival was specified as the primary clinical endpoint, referring to the time period between the date of diagnosis and the date when a new event associated with the cancer—such as progression, local recurrence, distant metastases or death—occurred.

### Preprocess of DNA methylation data

Preprocess of the DNA methylation data was essential before the statistical analyses and predictive model establishment. First of all, we counted the number of methylation sites with not available (NA) beta value and removed the sites with over 10% not available value. The remaining NA data was assumed with ‘impute.knn’ function from impute package^15^. Then, the methylation β values were normalized using the ‘betaqn’ function from the wateRmelon package^16^. All the samples were divided into with-progression and without-progression group, and the methylation sites with significantly different levels between the two groups were identified based on the M value by using the ‘dmpFinder’ function in the minfi package^17^.

### Identification of the CpG sites-derived risk signature

The univariate Cox proportional hazard analysis was implemented in the training cohort to screen methylation sites that are significantly related to TGCT patients’ PFS. Then, the lasso Cox regression analysis was performed using the ‘glmnet’ R package to screen the key methylation sites affecting the PFS of TGCT. Subsequently, key methylation sites from lasso analysis were further included in the multivariate Cox regression analysis. Finally, the risk score for every patient was calculated as follows: risk score = $\sum {(\upbeta_{{\text{i}}}* {\text{coef}}_{{\text{i}}} {)}}$(‘i’ = the number of prognostic methylation sites, ‘β_i_’ represents the beta value of each methylation site, ‘coef_i_’ represents the coefficient of each methylation site. Then, TGCT patients were divided into high-risk and low-risk groups according to the median score. The differences in PFS between the high-risk and low-risk groups were analyzed using Kaplan–Meier (K–M) method using the public R package ‘survival’^18^. A receiver operating characteristic (ROC) curves were used to evaluate the risk score model performance using the ‘survivalROC’ package. The differences in risk score among different clinicopathological groups were compared and visualized.

### Functional enrichment analysis

Transcriptome data of the TCGA–TGCT cohort from the TCGA database were retrieved and analyzed for differential expression between different risk groups using the limma package^19^. Differentially expressed genes were screened by adjusted p-value < 0.05 and |logFC| > 1. Gene Ontology (GO) and Kyoto Encyclopedia of Genes and Genomes (KEGG) pathway^20–22^ enrichment analyses were performed using the clusterprofiler package^23^, and terms were identified as significantly enriched while an adjusted p-value < 0.05 was achieved.

### Immune infiltration and chemosensitivity

CIBERSORT is an algorithm utilizing the expression values of 547 genes to assess the composition of immune cells in tissues. Immune infiltration of the 22 immune cell types in the high- and low-risk groups of the TCGA–TGCT cohort was determined and compared using the CIBERSORT package based on TCGA–TGCT cohort transcriptome data^24^. The differences in chemotherapy sensitivity between high- and low-risk groups were evaluated using the pRRophetic package^25^.

### Consensus clustering analysis

Consensus clustering was performed to identify a novel PFS-related CpG sites-based classification via the ‘ConsensusClusterPlus’ R package^26^. Then, the K–M curve was conducted to appraise the PFS of different CpG site-derived clusters, and differences in risk scores among clusters were compared and visualized.

### Construction and validation of the nomogram

A nomogram was constructed using the ‘rms’ R package after identifying independent risk factors of PFS for TGCT patients^27^. The univariate Cox proportional hazard analysis and multivariate Cox proportional hazard analysis were performed to identify the independent prognostic factors of TGCT patients’ PFS. C-index and calibration plots were executed to weigh the predictive performance of the established nomogram. Decision curve analysis (DCA) was performed using the rmda package to compare the benefit of all strategies in PFS prediction.

### Statistical analysis

All statistical analyses were conducted by the R software version 4.2.0 and SPSS software version 12. Univariate and multivariate Cox regression analyses were conducted to identify independent risk factors for PFS prediction of TGCT. All statistical p values are two-sided and p < 0.05 represents statistical significance.

## Results

### Clinical characteristics of the study populations

**Figure 1:**
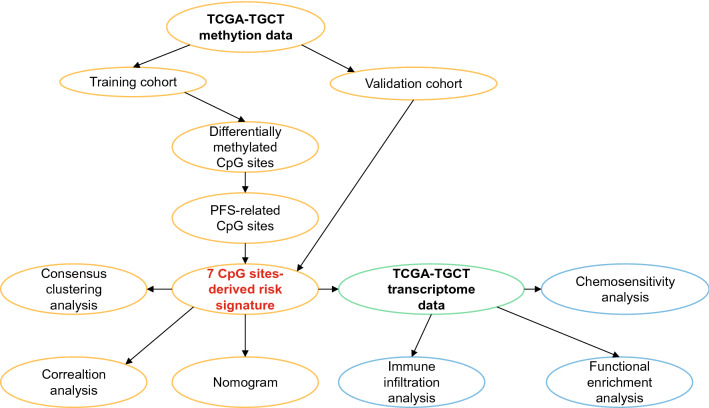
Flowchart of the present study.

In total, 128 TGCT patients with complete methylation and survival data were included in this study. The median age at diagnosis was 31 years (range, 18–67). Clinical stage of TGCT patients ranged from I to IS, with 42.97% (n = 55) in stage I, 10.94% (n = 14) in stage II, 10.16% (n = 13) in stage III, and 35.94% (n = 46) in stage IS. Of these patients, 52.34% (n = 67) had seminoma and 47.66% (n = 61) had non-seminoma histology. Lymphovascular invasion was present in 42.97% (n = 55) of patients, and 53.91% (n = 69) and 19.53% (n = 25) had received chemotherapy and radiotherapy treatments, respectively. Serum markers were divided into five groups according to the serum level of LDH, hCG, and AFP: S0 (31.82%, n = 41), S1 (28.03%, n = 37), S2 (25.76%, n = 33), S3 (3.79%, n = 5), and SX (9.09%, n = 12). The number of patients with and without progression was 35 (27.34%) and 93 (72.66%), respectively. (Table 1). All patients were randomly divided into the training cohort (89 patients) and the validation cohort (39 patients). Figure 1 showed the overall design and flowchart of the present study.

**Table 1:** Clinical characteristics of included patients.

Characteristics	Total (n = 128)		Training dataset (n = 89)		Testing dataset (n = 39)	
	n	%	n	%	n	%
Age						
≥ 31	69	53.91	47	52.81	22	56.41
< 31	59	46.09	42	47.19	17	43.59
Histology						
Seminoma	67	52.34	49	55.06	18	46.15
Non-seminoma	61	47.66	40	44.94	21	53.85
Lymphatic vascular infiltration						
Yes	55	42.97	39	43.82	16	41.03
No	73	57.03	50	56.18	23	58.97
Chemotherapy						
Yes	69	53.91	39	43.82	30	76.92
No	59	46.09	50	56.18	9	23.08
Radiotherapy						
Yes	25	19.53	16	17.98	9	23.08
No	103	80.47	73	82.02	30	76.92
Adjuvant therapy						
Yes	80	62.50	54	60.67	26	66.67
No	48	37.50	35	39.33	13	33.33
Stage						
I	55	42.97	41	46.07	14	35.90
II	14	10.94	7	7.87	7	17.95
III	13	10.16	6	6.74	7	17.95
IS	46	35.94	35	39.33	11	28.21
Serum markers						
S0	41	32.03	30	33.71	11	28.21
S1	37	28.91	27	30.34	10	25.64
S2	33	25.78	21	23.60	12	30.77
S3	5	3.91	2	2.25	3	7.69
SX	12	9.38	9	10.11	3	7.69
Survival status						
With progression	35	27.34	26	29.21	9	23.08
Without progression	93	72.66	63	70.79	30	76.92

### Identification of methylation signature associated with PFS

**Figure 2:**
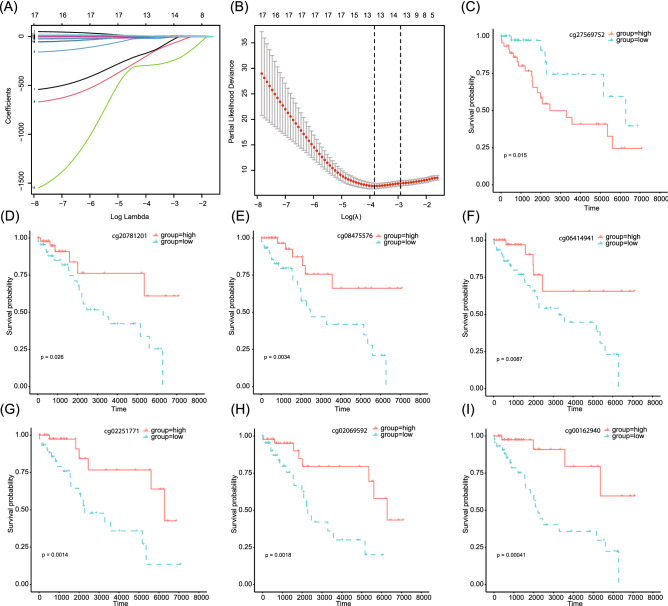
Identification of independent prognostic CpG sites. (**A**) lasso coefficient profiles of the methylation sites. A coefficient profile plot was produced against log(lambda) sequence. (**B**) Ten-fold cross-validation for selection of the parameter λ. (**C**–**I**) K–M survival analysis of cg00162940, cg02069592, cg02251771, cg06414941, cg08475576, cg20781201, cg27569752 in the TCGA–TGCT cohort, respectively. The cohort was separated into high and low groups according to the median β value of each CpG site.

Between the without-progression group and the with-progression group, we identified a large number of differential methylation sites (86,665 sites, p < 0.05; 28,174 sites, p < 0.01). To narrow the scope, we carried out the subsequent analysis of 2268 differential methylation genes with p values < 0.001. Univariate Cox regression analysis found that 1472 differential methylation positions were significantly correlated with PFS (p < 0.05). Seventeen sites with p values < 0.0005 were selected for lasso and multivariate Cox regression analysis and eventually 7 independent prognosis-related CpG sites were obtained, namely cg00162940, cg02069592, cg02251771, cg06414941, cg08475576, cg20781201 and cg27569752 (Fig. 2A,B). According to the median of β value of each site, 128 TCGA–TGCT individuals were divided into hypermethylation and hypomethylation groups, and K–M analysis was carried out. The results showed that these methylation sites were significantly correlated with the PFS of TGCT, among which cg27569752 hypermethylation predicted poor PFS, while hypermethylation of other sites predicted better PFS (Fig. 2C–I). As shown in Table 2, these positions are located in regions near 5 genes (PPM1D, PANX1, ENDOD1, MAF, MYH2), 1 DNase-I-hypersensitive site (DHS) region, and 3 enhancer regions.

**Table 2:** Overview of included CpG sites in terms of location, gene annotation, and gene function of the 11 CpG-sites in the risk signature.

CpG sites	Chromosome	Strand	Gene	Feature	CGI	DHS	Enhancer
cg00162940	17	F	PPM1D	TSS1500	Shore	NA	NA
cg02069592	14	R		IGR	Opensea	TRUE	TRUE
cg02251771	11	R	PANX1	Body	Opensea	NA	NA
cg06414941	11	F	ENDOD1	3'UTR	Opensea	NA	NA
cg08475576	16	R	MAF	TSS1500	Island	NA	NA
cg20781201	11	F		IGR	Opensea	NA	TRUE
cg27569752	17	F	MYH2	5'UTR	Opensea	NA	TRUE

*CGI* CpG island, *DHS* DNase-I-hypersensitive sites, *PPM1D* protein phosphatase, Mg^2+^/Mn^2+^ dependent 1D, *PANX1* pannexin 1, *ENDOD1* endonuclease domain containing 1, *MAF* MAF bZIP transcription factor, *MYH2* myosin heavy chain 2, *TSS200* transcription start sites, *IGR* intergenic region, *5*′*UTR* 5′-untranslated region, *3*′*UTR* 3′-untranslated region.

### Construction of CpG-derived risk model

**Figure 3:**
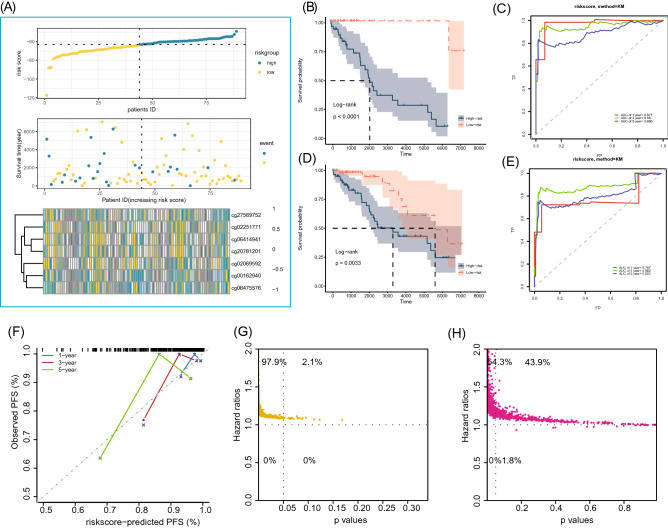
Construction and validation of CpG sites-derived risk score model. (**A**) Distribution of the risk score, survival status, and methylation status of included CpG sites in the training cohort. (**B**) K–M curves of high- and low-risk groups in the training cohort; (**C**) receiver operating characteristic curve of the risk score in predicting the 1-, 3-, and 5-PFS in the training cohort. (**D**) K–M curves of high- and low-risk groups in the entire cohort; (**E**) receiver operating characteristic curve of the risk score in predicting the 1-, 3-, and 5-PFS in the entire cohort. (**F**) The calibration curve of the risk score on the predicted PFS was obtained by self-sampling the entire cohort for 1000 times (39 samples each). (**G**,**H**) The HR and p-value distributions of the risk score in predicting PFS by randomizing the entire cohort 1000 times (at a ratio of 7:3).

Based on the seven PFS-related methylation loci identified above, a risk score model was built with the following formula: Risk score = − 345.765933*cg00162940 − 21.215846*cg02069592 − 9.555835*cg02251771 − 39.638966*cg06414941 − 575.141219*cg08475576 + 10.56158*7cg20781201 + 27.493894*cg27569752. The 128 patients in the TCGA cohort were divided into high-risk and low-risk groups according to the median of risk score (Fig. 3A). The K–M survival analysis showed significant differences in PFS between the high- and low-risk groups, with poorer PFS in the high-risk group and better PFS in the low-risk group, which could be verified in both the training cohort and the whole cohort. The ROC analysis showed that the risk score model had better performance in predicting 1, 3, and 5-year PFS of TGCT (Fig. 3B–E). Due to the limited samples in the training cohort, its verification effect was not ideal (data not provided). To further assess the relationship between the risk model and PFS and its effectiveness in predicting PFS, we used the self-sampling validation method and selected 30% (39 cases) of the samples for prediction each time, as shown in Fig. 3F. Due to the limited number of samples, its prediction performance is generally poor, suggesting that more available sample data are urgently needed in the prognosis research of TGCT. Through 1000 random grouping (at a ratio of 7:3) and prognostic analysis, the incidence probability of risk score significantly correlated with PFS was 97.9% and 54.3% in the high-sample group (70% of samples) and the low-sample group (30% of samples), respectively, indicating that the insufficient sample size might increase the accuracy of predicting PFS (Fig. 3G,H).

### The relationships between risk score and clinicopathological characteristics

**Figure 4:**
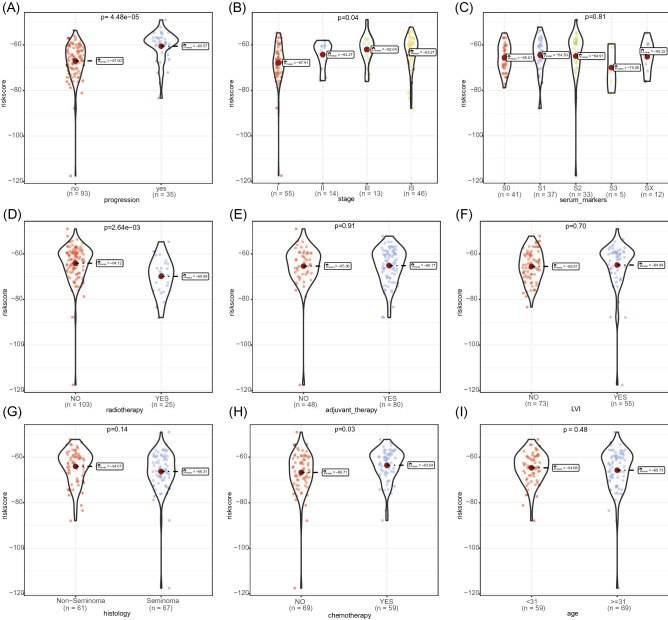
Distributions of the risk score in different clinicopathological groups, including (**A**) survival status, (**B**) stage, (**C**) serum markers, (**D**) radiotherapy, (**E**) adjuvant therapy, (**F**) lymphatic vascular infiltration (LVI), (**G**) histology, (**H**) chemotherapy, and (**I**) age.

To elucidate the relationship between risk scores and clinical pathological features, we compared the risk scores among different prognosis status, stage, serum marker, radiotherapy, chemotherapy, adjuvant therapy, lymphatic vascular infiltration (LVI), histology, and age groups (Fig. 4). The results showed that there were significant differences in risk scores among different prognosis status, stage, chemotherapy, and radiotherapy groups. The cases with progression had higher risk scores than those without progression, and the higher the stage, the higher the risk score. Patients who received radiotherapy had lower risk scores than those who did not receive radiotherapy. Conversely, patients who received chemotherapy had higher risk scores than those who did not receive chemotherapy. Furthermore, the methylation levels of these CpG sites in different survival status, stage, chemotherapy, and radiotherapy were compared (Figs. S1–S4). It was observed that several CpG sites had drastically different methylation levels between groups.

### Functional enrichment analysis

**Figure 5:**
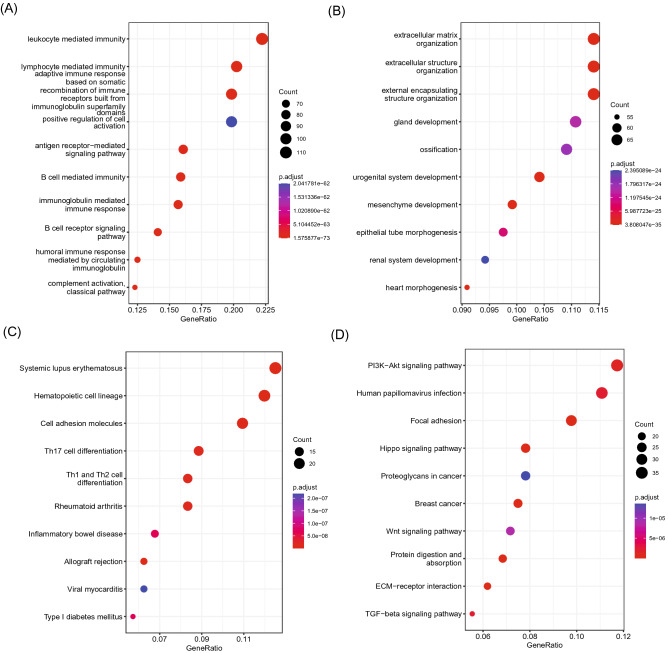
Functional enrichment analysis of the differentially expressed genes between high- and low-risk groups. (**A**) The top ten enriched GO terms of the upregulated genes with a higher gene count. (**B**) The top ten enriched GO terms pathways of the downregulated genes with a higher gene count. (**C**) The top ten enriched KEGG pathways of the upregulated genes with a higher gene count. (**D**) The top ten enriched KEGG pathways of the downregulated genes with a higher gene count.

The transcriptome data of 128 TCGA–TGCT patients were obtained from the TCGA database and analyzed for differential expression and enrichment (Fig. 5). Through these analyses, we identified 1452 genes (p.adj < 0.05 and |logFC| > 1) that differentially expressed between high- and low-risk groups, in which 666 genes were significantly up-regulated, and 786 genes were significantly down-regulated. Specifically, higher-expressed genes were found to be significantly enriched in immunity-related biological processes, and and hematopoietic cell lineage/T cell differentiation pathways, whereas lower-expressed genes were significantly enriched in the biological processes involved in extracellular matrix organization and associated with the PI3K-AKT signaling pathway, focal adhesion, hippo signaling pathway, Wnt signaling pathway, protein digestion/absorption pathways.

### Immunoinfiltration and chemotherapy sensitivity

**Figure 6:**
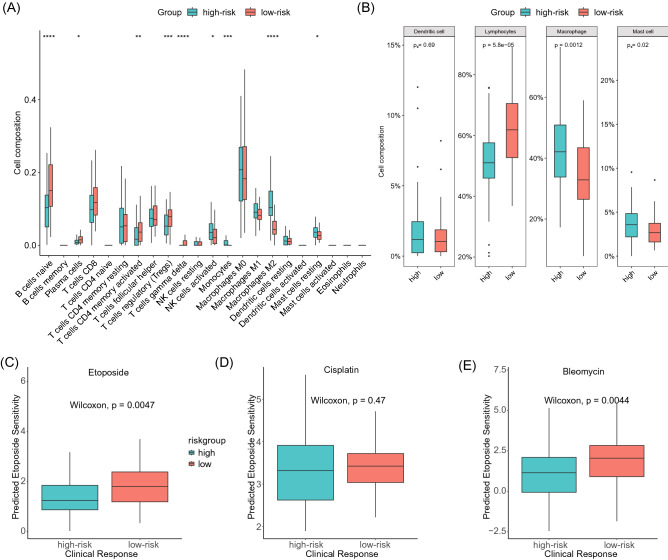
Immunoinfiltration and chemotherapy sensitivity analysis in the TCGA–TGCT cohort. (**A**) Comparison of the infiltration of 22 types of immune cells in high-risk and low-risk groups. (**B**) Comparison of the infiltration of 4 categories of immune cells in high-risk and low-risk groups. (**C**–**E**) Comparison of chemosensitivity to etoposide, cisplatin, and bleomycin in high-risk and low-risk groups, respectively.

Further analysis of the immune infiltration and chemosensitivity between different risk groups was conducted. We found that, out of the 22 types of immune cells, 16 were ubiquitously present in the TGCT cohort, and 9 of them exhibited significantly different infiltration levels between different risk groups. Specifically, the infiltration levels of activated NK cells, monocytes, M2 macrophages, and resting mast cells in the high-risk group were significantly higher than those in the low-risk group. Conversely, the infiltration levels of naive B cells, plasma cells, activated CD4 memory T cells, regulatory T cells, and gamma delta T cells in the high-risk group were significantly lower than those in the low-risk group (Fig. 6A). Further grouping of the 22 cells into dendritic cells, lymphocytes, macrophages, and mast cells revealed that the infiltration level of lymphocytes in the high-risk group was significantly higher than that in the low-risk group, while the infiltration level of macrophages was significantly lower than that in the low-risk group (Fig. 6B). Subsequent assessment of the chemosensitivity between high- and low-risk groups using the three most commonly used chemotherapeutic drugs in the TCGA–TGCT cohort, namely etoposide, cisplatin, and bleomycin, indicated that the low-risk group was more sensitive to etoposide and bleomycin than the high-risk group (Fig. 6C–E).

### Prognostic CpGs-derived clusters

**Figure 7:**
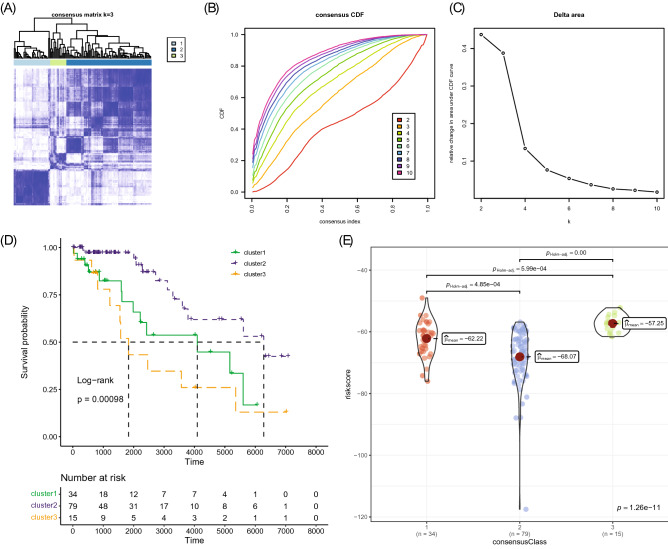
Consensus clustering analysis of the TCGA–TGCT cohort based on the 7 CpG sites. (**A**) The consensus score matrix of all samples when k = 3 in the TCGA–TGCT cohort. (**B**) Consensus values range from 0 to 1. (**C**) The corresponding relative change in area under the cumulative distribution function (CDF) curves when cluster number changes from k to k + 1. (**D**) K–M curves of the three clusters in the TCGA–TGCT cohort. (**E**) Comparison of the risk score among the three clusters.

We employed a consensus clustering analysis of 128 TCGA–TGCT cohorts by seven CpG sites related to PFS. Considering the clustering performance and sample size, 128 samples were divided into three clusters (Fig. 7A–C). K–M survival analysis showed that the PFS of these three clusters significantly differed, with cluster 2 having the best prognosis followed by cluster 1 and then cluster 3 having the worst (Fig. 7D). Comparisons of the risk score distributions among different clusters in Fig. 7E showed that the risk score distributions significantly differed among clusters, with the risk score of cluster 3 being significantly higher than those of the other two clusters, and the risk scores of cluster 2 being significantly lower than those of the other two clusters.

### Nomogram development and assessment

**Figure 8:**
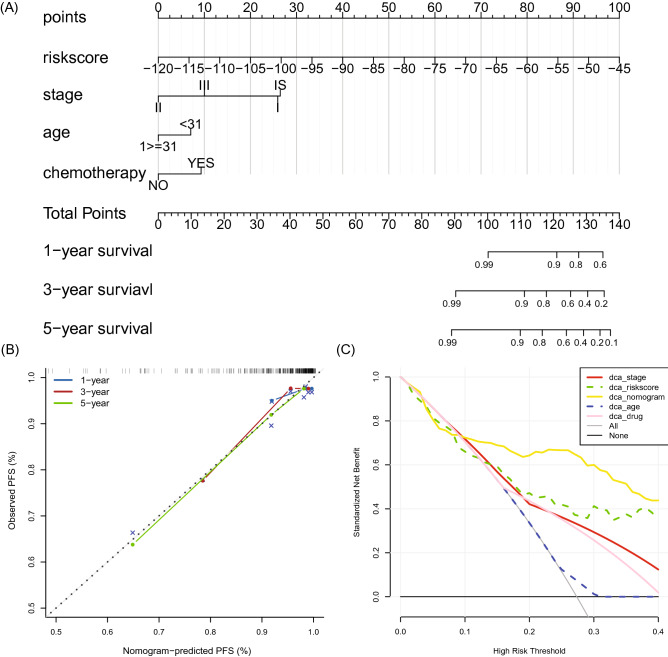
Construction and evaluation of a nomogram for the prediction of TGCT patients' PFS. (**A**) The nomogram was constructed by combing the risk score, stage, age, and chemotherapy. (**B**) calibration curves of the nomogram in predicting the 1-, 3- and 5-year PFS. The closer the dotted line fit to the ideal line, the better the predictive accuracy of the nomogram is. (**C**) Decision curve analysis of the nomogram and other risk strategies in predicting the PFS of TGCT.

Univariate Cox survival analysis revealed that the clinical stage and the risk score were prognostic factors for PFS of TGCT patients. Subsequently, results of multivariate Cox regression analysis indicated that the risk score (p < 0.001), age (p = 0.035), chemotherapy (p = 0.012), and clinical stage (p = 0.006) were significantly associated with TGCT patients' PFS (Table 3). Based on these results, a nomogram was constructed that incorporated the risk score model, age, stage, and chemotherapy, providing a reliable predictive tool with a C-index of 0.812 in the entire cohort. (Fig. 8A). The calibration curves exhibited a good predictive accuracy (Fig. 8B), with decision curve analysis suggestive of the superior performance of the prediction model compared to alternative strategies (Fig. 8C). Collectively, our results indicate the established nomogram provides an effective tool for predicting the PFS of TGCT patients.

**Table 3:** Univariate Cox regression analysis and multivariate Cox regression analysis outcome based on methylation risk score and other clinical factors.

Factors	Univariate Cox analysis				Multivariate Cox analysis			
	p-value	HR	LCI	UCI	p-value	HR	LCI	UCI
Age	0.472	0.784	0.403	1.523	0.035	0.452	0.216	0.946
Histology	0.571	0.822	0.417	1.621				
LVI	0.395	0.738	0.367	1.486				
Chemotherapy	0.311	1.452	0.706	2.989	0.012	2.847	1.257	6.446
Radiation	0.577	0.795	0.356	1.779				
Adjuvant therapy	0.805	1.112	0.477	2.591				
Stage (I as reference)	0.02				0.006			
II	0.073	0.153	0.02	1.192	0.007	0.054	0.006	0.449
III	0.282	0.436	0.096	1.98	0.026	0.166	0.034	0.808
IS	0.113	1.844	0.865	3.932	0.884	1.063	0.467	2.417
Serum markers (S0 as reference)	0.099							
S1	0.265	1.792	0.643	4.997				
S2	0.011	3.357	1.316	8.563				
S3	0.23	3.751	0.433	32.477				
SX	0.873	1.19	0.141	10.053				
Risk score	< 0.001	1.17	1.079	1.27	< 0.001	1.162	1.075	1.257

*HR* hazard ratio, *LCI* lower 95% confidence interval, *UCI* upper 95% confidence interval, *LVI* lymphatic vascular infiltration.

## Discussion

TGCT is the most prevalent tumor in young adults, having persisted in rising for the past several decades in most populations^28^. Although the mortality rate of TGCT has improved, 20–30% of patients have shown resistance to traditional chemotherapy, with some undergoing refractory disease^29^. Currently, the dependability of traditional clinicopathological parameters, such as TNM staging and serum biomarkers, should be enhanced in order to more precisely predict the prognosis of TGCT. A variety of molecular markers have been developed to forecast the prognosis in various tumors, and the application of DNA methylation as a prognostic biomarker has a few merits over other molecular biomarkers, including higher stability^30^, smaller sample size requirement^31^, and relative higher accuracy^32^. Evidentiary support has demonstrated that DNA methylation signatures had achieved satisfactory results in the prognostic prediction of multiple types of cancer. For example, a 13-DNA methylation signature was discovered to yield a high evaluative performance in the RFS prediction in stage I lung cancer^11^. Another recent study revealed that a 6-DNA methylation signature displayed a better value for predicting recurrence-free survival of thyroid papillary cancer^12^. In gastric cancer, Ma et al. found that DNA methylation signature performed well in prognostic prediction and established a nomogram model based on 11-DNA methylation sites and clinicopathological indicators^13^. However, a quantitative method to predict a TGCT patient’s probability of PFS based on DNA methylation signature has yet to be developed.

By analyzing whole-genomic methylation profiles in 128 TGCT samples, we found that 7 DNA methylation sites were related to PFS in patients with TGCT. The 7-DNA methylation signature was capable to distinguish patients with low- or high-risk, and serving as an independent factor for TGCT patients' PFS after adjusting for the effects of clinical indicators. A previous study successfully established a nomogram with good predictive performance on the basis of a five-gene signature and four clinical factors (age, serum marker, lymphovascular invasion, and histological types) in a nomogram. In this study, we included a DNA methylation signature and three clinical factors (stage, age, and chemotherapy) in a nomogram, which yielded a better benefit in PFS prediction of TGCT when compared to these factors used individually.

The 7 CpG sites identified in this study were associated with five genes: PPM1D, PANX1, ENDOD1, MAF, and MYH2. PPM1D is a tumor suppressor gene and has been associated with various types of cancer, including breast, ovarian, and colorectal cancer; mutations of this gene may impact the ability of the body to repair damaged DNA^33^. PANX1 encodes a protein involved in intercellular communication, and mutations of this gene are associated with an increased expression of molecules involved in cancer growth^34^. ENDOD1 encodes a protein that is implicated in cell death, and is found at elevated levels in certain types of cancer^35^. MAF is a gene involved in cell proliferation and has been observed to be mutated frequently in various types of cancer^36^. MYH2 encodes a protein involved in DNA repair, and is associated with colorectal cancer^37^. Overall, further research is needed to fully understand the relationship between these genes and cancer.

It was revealed that differentially expressed genes between different risk groups were associated with processes of immunity and extracellular matrix organization. Recent studies have suggested that a higher level of immunity may improve the prognosis of TGCT cancer^38,39^. Therefore, it is important for patients to maintain an adequate level of immunity to enhance their chances of positive outcomes. In addition, it was demonstrated that differentially expressed genes between different risk groups were primarily enriched in T cell differentiation and multiple crucial signal transduction pathways, such as PI3K-AKT, Hippo, and Wnt signaling pathways. The differences in these biological processes and pathways may be the underlying cause for the significantly different PFS between different risk groups.

The association between immune response and prognosis for TGCT has been extensively studied in recent years^40^. Results from several studies suggest that patients with higher levels of immune cells such as CD4 and CD8 T lymphocytes, natural killer cells, and monocytes, have a better prognosis than those with lower levels^41^. Furthermore, there is evidence that these immune cells can be used to predict and classify the aggressiveness of TGCT tumors and that they may also have the therapeutic potential^42^. In this study, higher lymphocytes infiltration and lower macrophage infiltration were observed in the low-risk group as compared to the high-risk group. T cell infiltration and tumor-infiltrating lymphocytes have been associated with a favorable prognosis in TGCT. Moreover, the presence of T cells in the tumor microenvironment has been linked to a better response to chemotherapy and better overall outcome^43^. Our results also showed that the low-risk group with higher T cell infiltration had greater sensitivity to etoposide and bleomycin chemotherapy. These results suggest that the CpG-based risk model has strong predictive capabilities in terms of both immune infiltration and chemotherapeutic drug sensitivity, which may also play an important role in the current focus on immune therapy response but requires further analysis.

Apart from the inspiring results, there are also several limitations in our study. Firstly, the 7-DNA methylation signature was identified from the TCGA database, lacking of the external validation cohort. This may generate a hazard of selection bias. Secondly, the high cost of methylation tests limit their clinical application, but this is being resolved with the advancement of technology. Despite the above-mentioned limitations, our study still provided some valuable implications. Firstly, employing the lasso method to identify PFS-related methylation sites in the study solved the multicollinearity problem and generated more reliable results. Secondly, the 7-DNA methylation signature of TGCT was capable to separate TGCT patients into high- and low-risk groups and predicted PFS with robust accuracy. Moreover, the established nomogram by integrating clinical indicators and methylation signature provided a quantitative method for accurate PFS prediction of TGCT patients, which will contribute to the development of the field of personalized medicine for TGCT.

## Conclusion

In this study, we identified a 7-DNA methylation signature as an independent prognostic biomarker for predicting the PFS of TGCT patients and constructed a risk model based on the 7-DNA methylation sites to discriminate high- and low-risk TGCT patients. The CpG site-derived risk model was associated with various processes and pathways including immunity, extracellular matrix organization, T cell differentiation, and multiple signal transduction pathways. Meanwhile, significant differences were observed in immune infiltration and chemosensitivity between different risk patients, which might contribute to the prognosis of TGCT. A nomogram that integrated the 7-DNA methylation signature, age, stage, and chemotherapy was also established with satisfactory performance to predict PFS of TGCT. Our results shed light on the methylation biology of TGCT and promote the development of effective prognostic biomarkers for TGCT.

## Supplementary Information

Supplementary Figure S1.

Supplementary Figure S2.

Supplementary Figure S3.

Supplementary Figure S4.

## Data Availability

The data that support the fndings of this study are available from the Cancer Genome Atlas (TCGA, http://cancergenome.nih.gov/).
